# Sleep position and risk of late stillbirth

**DOI:** 10.1186/1471-2393-12-S1-A12

**Published:** 2012-08-28

**Authors:** Tomasina Stacey, Edwin A Mitchell

**Affiliations:** 1 University of Auckland, New Zealand; 2 University of Leeds, UK

## 

Obesity is an established risk factor for stillbirth, however the reason for this is not known. There are several possible mechanisms which have not previously been investigated, such as maternal and fetal hypoxia from adverse maternal sleep position or sleep disordered breathing (SDB). We hypothesised:

1. that obesity is an independent risk factor for stillbirth,

2. that after adjustment for established risk factors for stillbirth, the higher rates in Maori and Pacific Island babies are no different to that of European babies, and

3. that sleep disordered breathing is a risk factor for stillbirth, and that this (at least in part) explains the increased risk with obesity.

The Auckland stillbirth study was a case control study conducted in Auckland, New Zealand between 2006 and 2009 [1]. In this study, we explored potentially modifiable risk factors for late stillbirth, including maternal position on going to sleep [2]. We found that maternal non-left position on going to sleep (on the last night prior to stillbirth, or prior to interview) was associated with a two fold increase in late stillbirth adjusted odds ratio (aOR) 2.0 (95% confidence interval (CI): 1.2 to 3.3) [2]. The greatest effect was when the mother went to sleep on her back (aOR 2.5, 95% CI: 1.0 to 6.2) and intermediate when on the right (aOR 1.7 95% CI: 1.0 to 3.0). These findings remained significant after adjustment for known confounders such as maternal body mass index, age and smoking. Although we could not establish an association between SDB (measured using self reported snoring and daytime sleepiness) and risk of stillbirth, these symptoms are common in pregnancy and may not identify true SDB.

This is the first time that an association between maternal sleeping position and risk of late stillbirth has been described and therefore the finding should be interpreted with some caution until further studies have confirmed or refuted it. There is, however, some evidence that may support the biological plausibility of such an association, as maternal body position has been found to impact on maternal and fetal physiological parameters. Specifically it has been shown that maternal cardiac output in late pregnancy is greatest in the left lateral position, intermediate in the right lateral position and lowest when the mother is supine [3]. Similar graded effects have been found between fetal oxygenation in labour and maternal position, with optimum oxygen levels recorded with the mother on her left side [4]. This is speculated to be due to the anatomy of the lower abdomen and the potential compression of the aorta and inferior vena cava caused by the weight of the uterus and growing fetus when the woman is in either the supine position or in the right lateral position.

The absolute risk of late stillbirth for women who went to sleep on their left side was 1.96/1000 and was 3.93/1000 for women who did not go to sleep on their left. This study identified a potentially modifiable risk factor for stillbirth, but confirmatory studies are needed before public health recommendations can be made.
